# Combined Effects of Sulfhydryl-Grafted Palygorskite and Manganese Fertilizers in Reducing Cadmium Accumulation in Wheat

**DOI:** 10.3390/plants15040621

**Published:** 2026-02-15

**Authors:** Xingru Wang, Zhijun Liu, Xuefeng Liang, Xichao Sun, Yuebing Sun, Qingqing Huang

**Affiliations:** 1 Agro-Environmental Protection Institute, Ministry of Agriculture and Rural Affairs, Tianjin 300191, China; 2 Hebei Huakan Resources and Environment Survey Co., Ltd., Chengde 067000, China

**Keywords:** cadmium, manganese, sulfhydryl-grafted palygorskite, immobilization, wheat

## Abstract

Cadmium (Cd) contamination in alkaline soils poses a significant threat to wheat production and food safety. This study investigated the combined effects of sulfhydryl-grafted palygorskite (SGP) and manganese fertilizers (MnO and MnSO_4_) on Cd immobilization in soil and its subsequent accumulation in spring wheat via a pot experiment. The results demonstrated that the combined application of SGP and MnSO_4_ exhibited the highest efficiency, reducing grain Cd concentration by 62.5% compared to the control, which was superior to the single SGP treatment (39.5% reduction). Simultaneously, grain Mn content increased markedly. Soil microbial community analysis confirmed the environmental safety of this strategy, as it showed no substantial shifts in bacterial diversity or community structure. The combined mechanism was attributed to a dual action: SGP served as the primary external immobilization strategy by sequestering Cd in the soil and diminishing its bioavailability, whereas Mn fertilizers, especially MnSO_4_, functioned as a restrictive mechanism within the plant, competing with Cd for uptake and transport pathways and modifying its translocation, ultimately restricting its allocation to the grain. This study provides a novel, efficient, and environmentally friendly dual strategy of immobilization and antagonism for the safe production of wheat in Cd-contaminated alkaline soil.

## 1. Introduction

Heavy metal pollution in agricultural soils has emerged as a serious global environmental concern in recent years. In China, the 2014 National Soil Pollution Status Survey Bulletin revealed that 19.4% of agricultural soil samples failed to meet environmental quality standards. Notably, 82.8% of these exceedances were due to heavy metals and metalloids. Among these, cadmium (Cd) was the most prevalent and had the highest exceedance rate (7.0%) [1]. As a highly toxic element, Cd not only inhibits plant growth but enters the food chain via plant uptake from contaminated soil. This pathway ultimately leads to Cd accumulation in the human body, causing various detrimental health effects, including itai-itai disease, organ dysfunction, and cancers [2]. Wheat (*Triticum aestivum* L.) is a major staple grain in China [3] and a significant source of dietary Cd intake, especially in northern China [4]. Currently, wheat Cd contamination in northern China farmlands is expanding and intensifying [5,6]. Therefore, developing strategies to reduce Cd accumulation in wheat grain is imperative for food safety and human health.

In recent years, numerous strategies have been developed to achieve the safe utilization of Cd-contaminated farmland by remediating soil and reducing Cd accumulation in crop edible parts. Key approaches include cultivating low-Cd-accumulating cultivars [7], implementing water management [8], applying foliar sprays [9], and employing chemical immobilization [10]. Among these, chemical immobilization using soil amendments is widely favored due to its high efficiency, simplicity, and cost-effectiveness [11]. Currently, a diverse range of materials are commonly applied to Cd-polluted farmland. These include inorganic amendments like lime [12] and clay minerals (e.g., sepiolite [13] and zeolite [14]), as well as organic materials like biosolid [15], biochar [16], and humic acids [17]. For example, Chen et al. [18] reported that sepiolite application reduced Cd concentration in rice grain by 47–49%, with immobilization effects persisting for at least two years. While extensive research on chemical immobilization has focused on acidic soil, traditional pH-regulating amendments are inherently unsuitable for alkaline environments, which predominate in China’s major wheat-producing regions. Given these constraints, effective remediation in alkaline soils requires innovative amendments driven by direct chemical mechanisms rather than those relying on soil pH modification. Among various candidates, sulfhydryl-grafted palygorskite (SGP), a novel and high-performance amendment, has proven effective for Cd immobilization in both acidic soil and alkaline soil, significantly reducing Cd accumulation in rice and wheat grains [19,20,21]. For instance, our previous research showed that SGP application significantly immobilized Cd in in situ field experiments, achieving an immobilization efficiency of 40.1–61.5% and a reduction in wheat grain Cd concentration from 0.183 mg/kg to 0.056 mg/kg [20]. Additionally, SGP application also benefited wheat by increasing the relative abundance of plant growth-promoting rhizobacteria [22]. Collectively, these findings highlight that SGP has outstanding properties such as low application dose, high remediation efficiency, and environmental compatibility, establishing it a promising solution for Cd-contaminated alkaline wheat soil.

Manganese (Mn) is an essential micronutrient for plant growth and development. It activates at least 35 enzymes that sustain metabolic activity within various plant compartments [23]. Mn deficiency can reduce secondary metabolite levels, inhibit root development, and cause symptoms such as interveinal chlorosis, phosphorus deficiency, and iron toxicity, ultimately impairing crop growth and yield. In general, Mn deficiency is a widespread problem, particularly prevalent in dry, calcareous, and sandy soils [24]. In China, previous soil surveys revealed that 21.3% of cultivated land is latent Mn-deficient, with soil available Mn levels generally higher in the south than in the north. As a result, soil Mn bioavailability is low in the main wheat-producing areas of northern China, where Mn deficiency is quite common [25]. Soil or foliar application of Mn fertilizer are recommended strategies to mitigate Mn deficiency in the field [26,27]. Notably, Cd and Mn share comparable characteristics as divalent cations and exhibit similar antagonism during plant uptake and transport [28]. This relationship implies two key effects: (1) immobilization amendments targeting Cd might reduce soil Mn bioavailability due to their similar properties, potentially inducing Mn deficiency in crops, and, conversely, (2) Mn fertilization may enhance Mn accumulation while reducing Cd accumulation in plants due to their antagonistic interaction. For example, previous studies recorded that Mn fertilizers increased soil available Mn and plant Mn concentrations, while simultaneously decreasing Cd concentrations in wheat [21,29]. In light of these findings, it was hypothesized that combining Mn fertilizers with soil amendments would increase Mn accumulation and decrease Cd accumulation in plants. Given the demonstrated efficacy of SGP in the remediation of Cd-contaminated alkaline soil [19,20,21], we further propose that integrating SGP with Mn fertilizers provides a multi-stage protection strategy: SGP acts as the primary external sequestrant to immobilize Cd in the soil, while Mn functions as a restrictive mechanism to limit internal Cd translocation and simultaneously enhance plant Mn nutrition. To date, the combined potential of this integrated framework, particularly the comparative effectiveness of different Mn forms, has not been explicitly explored.

Building on the aforementioned arguments, the present study aimed to test the hypothesis that the combined application of SGP with Mn fertilizers suppresses Cd accumulation and enhances Mn accumulation in wheat grain using pot experiments with Cd-contaminated alkaline soil. Changes in soil properties, Cd and Mn mobility and fractionation, as well as their accumulation in wheat organs were monitored to assess the immobilization effectiveness of individual applications (SGP or Mn fertilizers) and their combined use. This work is expected to provide insights into developing practical solutions to concurrently mitigate Cd contamination and address Mn deficiency in wheat production systems.

## 2. Results

### 2.1. Changes in Soil Physicochemical Properties

**Figure 1:**
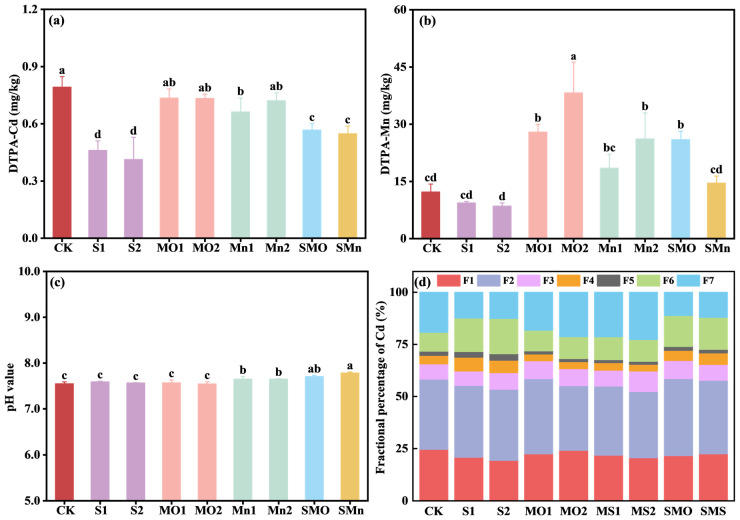
Effects of different treatments on soil DTPA-Cd (**a**), DTPA-Mn (**b**), pH value (**c**), and Cd chemical fractions (**d**). Treatment codes: CK, control; S1, 0.05% SGP; S2, 0.10% SGP; MO1, 0.05% MnO; MO2, 0.10% MnO; Mn1, 0.05% MnSO_4_; Mn2, 0.10% MnSO_4_; SMO, 0.05% SGP + 0.05% MnO; SMn, 0.05% SGP + 0.05% MnSO_4_. In panel (**d**), F1 to F7 represent the sequentially extracted Cd fractions: F1, exchangeable; F2, carbonate-bound; F3, Mn oxide-bound; F4, amorphous Fe oxide-bound; F5, crystalline Fe oxide-bound; F6, organic-bound; and F7, residual. The data are presented as means ± SDs (*n* = 3). Different letters among each treatment indicate significant differences as determined by the LSD test (*p* < 0.05).

Soil application of SGP alone or in combination with two Mn fertilizers significantly decreased DTPA-Cd content, with the single SGP treatment showing the best reduction effect, followed by the combined treatment (Figure 1a). When compared to the control group, the addition of SGP alone or in combination with two Mn fertilizers decreased DTPA-Cd by 41.9–47.8% and 28.4–30.8%, respectively. Specifically, applying Mn fertilizer alone had a limited effect on soil DTPA-Cd content compared to the control, with the exception of the Mn1 treatment, which resulted in a decrease of 7.16–16.4%. Regarding soil Mn availability, both the individual Mn application and its combination with SGP significantly increased the DTPA-Mn content, with the MO2 treatment exhibiting the most pronounced promotional effect (Figure 1b). Compared to the control, the addition of MnO and MnSO_4_ alone significantly increased DTPA-Mn by 128–212% and 50.8–114%, respectively, and the increases were more pronounced as application rates increased (Figure 1b). In addition, a single SGP treatment significantly decreased DTPA-Mn content by 23.5–30.1% when compared to the control. Furthermore, the combination treatments SMO (0.05% SGP + 0.05% MnO) and SMn (0.05% SGP + 0.05% MnSO_4_) increased DTPA-Mn by 112% and 18.9%, respectively, compared to the control, while remaining slightly lower than their respective single Mn treatments. In terms of soil pH, only single Mn treatments (Mn1 and Mn2) and the combined Mn fertilizers with SGP treatments (SMO and SMn) slightly increased soil pH by 0.10–0.24 units (Figure 1c).

As shown in Figure 1d, regardless of treatment, the majority of soil Cd exists in a carbonate-bound fraction (F2) (31.03–36.88%) and an exchangeable fraction (F1) (19.47–24.66%), which accounted for 66.5–72.3% of the total soil Cd in all treatments. Application of SGP and Mn fertilizer alone or in combination caused slight variations in the soil Cd fractions. For instance, S1, S2, SMO, and SMn treatments reduced F1 and F7 by 72.06–5.19% and 6.58–8.02% and increased F4 and F6 by 0.74–2.55% and 5.76–7.85%, while single Mn fertilizer treatments just slightly increased F3 by 0.29–2.47%. The above findings indicate that the Mn fertilizers alone did not alter the chemical speciation or mobility of Cd in this Cd-contaminated alkaline soil.

### 2.2. Analysis of Soil Microbial Diversity

**Figure 2:**
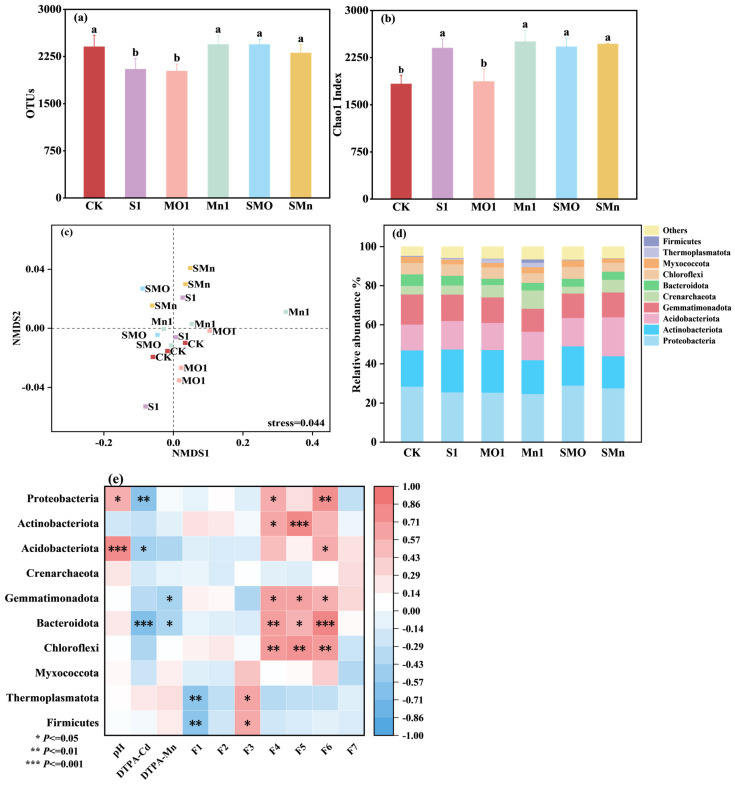
Effects of different treatments on soil bacterial communities: OTUs (**a**), Chao1 Index (**b**), Non-Metric Dimensional Scaling (NMDS) analysis (**c**), relative abundance of bacterial taxa at the phylum level (**d**), and correlation analysis between bacterial communities at the phylum level and soil physicochemical properties (**e**). CK, control; S1, 0.05% SGP; MO1, 0.05% MnO; Mn1, 0.05% MnSO_4_; SMO, 0.05% SGP + 0.05% MnO; SMn, 0.05% SGP + 0.05% MnSO_4_. In panel (**d**), F1 to F7 represent the sequentially extracted Cd fractions: F1, exchangeable; F2, carbonate-bound; F3, Mn oxide-bound; F4, amorphous Fe oxide-bound; F5, crystalline Fe oxide-bound; F6, organic-bound; and F7, residual. The data are presented as means ± SDs (*n* = 3). Different letters among each treatment indicate significant differences as determined by the LSD test (*p* < 0.05).

The application of SGP and Mn fertilizers, whether singly or in combination, did not induce a significant shift in the overall soil bacterial community structure. Figure 2a,b show the diversity indices of soil microbes under different treatments. The Chao1 indexes, reflecting species abundance, were not significantly affected by any of the amendments compared to the control (Figure 2a,b). Furthermore, Non-Metric Dimensional Scaling (NMDS) analysis based on Unifrac distance revealed no clear separation among treatments, indicating that the amendments did not significantly alter the overall bacterial community composition (Figure 2c).

At the phylum level, the dominant bacterial groups across all treatments included *Proteobacteria* (28.50%), *Actinobacteriota* (18.51%), *Acidobacteriota* (13.21%), *Gemmatimonadota* (15.40%), and *Crenarchaeota* (4.39%) (Figure 2d). Most treatments had minimal effects on the relative abundance of these major phyla. Notably, only the SGP + MnO treatment slightly increased the abundance of *Proteobacteria* by 2.02%, while other treatments reduced it by 2.09–13.16%. All treatments increased the abundance of *Acidobacteriota* (by 5.06–51.10%) but decreased that of *Gemmatimonadota* (by 12.03–23.43%) compared to CK. The MnSO_4_ treatment significantly enhanced the relative abundance of *Crenarchaeota*, which was 1.14 times that present in the control treatment.

Correlation analysis between soil properties and bacterial phyla revealed that pH was significantly positively correlated with *Proteobacteria* and *Acidobacteriota*, while soil DTPA-Cd was significantly negatively correlated with *Proteobacteria*, *Acidobacteriota*, and *Bacteroidota*. Soil DTPA-Mn showed a positive correlation with *Gemmatimonadota* and *Bacteroidota* and a negative correlation with *Crenarchaeota* (Figure 2e).

### 2.3. Cd Concentration in Different Wheat Organs

**Figure 3:**
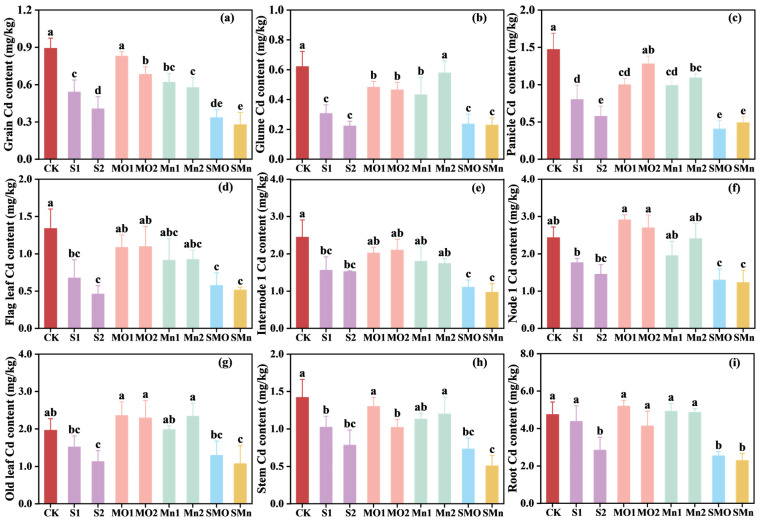
Effects of different treatments on Cd concentration in wheat organs: (**a**) grain, (**b**) glume, (**c**) panicle, (**d**) flag leaf, (**e**) internode 1, (**f**) node 1, (**g**) old leaf, (**h**) stem, and (**i**) root. CK, control; S1, 0.05% SGP; S2, 0.10% SGP; MO1, 0.05% MnO; MO2, 0.10% MnO; Mn1, 0.05% MnSO_4_; Mn2, 0.10% MnSO_4_; SMO, 0.05% SGP + 0.05% MnO; SMn, 0.05% SGP + 0.05% MnSO_4_. The data are presented as means ± SDs (*n* = 3). Different letters among each treatment indicate significant differences as determined by the LSD test (*p* < 0.05).

The concentration of Cd in different wheat organs was significantly influenced by the amendments (Figure 3). Single addition of SGP significantly reduced Cd concentrations across all organs, while co-application of SGP and Mn fertilizers further reduced wheat Cd concentrations. Regardless of treatments, Cd concentrations in various wheat organs showed the following pattern: root > node 1 > internode 1 > old leaf > panicle > stem > flag leaf > grain > glume. Furthermore, the effects of applying SGP alone and combining SGP and Mn fertilizers were consistent across all nine wheat parts, hence only the grain Cd data were considered in greater detail below. When compared to the control group, the addition of SGP alone significantly decreased grain Cd by 39.5%, and co-application of SGP and Mn fertilizers further reduced Cd concentration in grains by 17.31–31.55% compared to the single SGP treatment and by 62.5% compared to the control treatment, with co-application of SGP and MnSO_4_ (SMn) resulting in the lowest grain Cd concentration (Figure 3a). Apart from that, single addition of Mn fertilizers also significantly decreased grain Cd, with MnSO_4_ having a greater effect than MnO, as MnSO_4_ application significantly reduced Cd concentration in grains by 30.6–35.5% relative to the control treatment, while MnO application reduced Cd concentration in grains by 6.99% (Figure 3a). Furthermore, the measurement of grain dry weight revealed that none of the treatments significantly affected wheat productivity. Specifically, the grain yield per pot remained stable across all groups, ranging from 6.70 g to 6.93 g, confirming that the integrated application of SGP and Mn fertilizers at the tested dosages did not exert phytotoxic effects on wheat growth.

In addition, to investigate the effects of soil application of SGP alone or in combination with two Mn fertilizers on the Cd translocation and distribution in wheat plants, the correlations of Cd concentrations among different organs were analyzed for each treatment. As shown in Figure S1, distinct patterns of Cd distribution were observed under different amendment treatments. Soil application of SGP alone resulted in a comprehensive and significant positive correlation between grain Cd and Cd content in all other eight organs (Figure S1a), indicating a highly coordinated systemic distribution of Cd. In contrast, both Mn fertilizer treatments disrupted this pattern, with grain Cd showing significant positive correlations with Cd only in the glume, flag leaf, stem, and root (Figure S1b,c), suggesting a partial interruption of Cd co-translocation. Notably, co-application of SGP and Mn fertilizers re-established a full and significant positive correlation network between grain Cd and all other organs (Figure S1d).

### 2.4. Mn Concentrations in Different Wheat Organs

**Figure 4:**
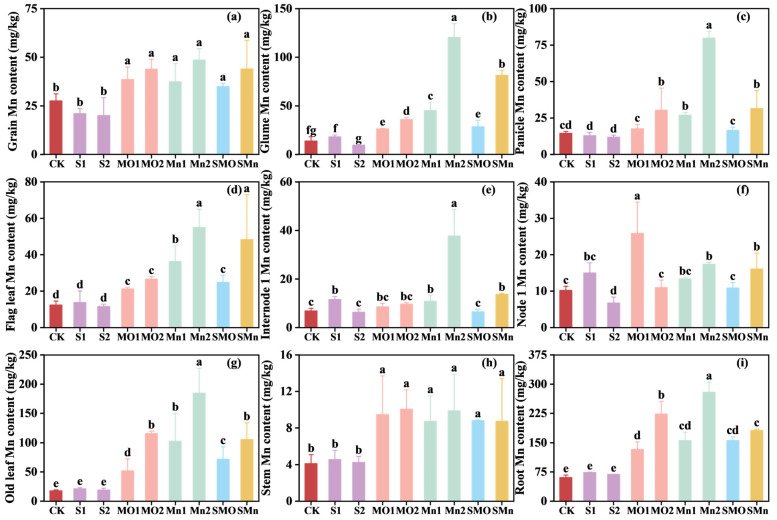
Effect of different treatments on Mn concentration in wheat organs: (**a**) grain, (**b**) glume, (**c**) panicle, (**d**) flag leaf, (**e**) internode 1, (**f**) node 1, (**g**) old leaf, (**h**) stem, and (**i**) root. CK, control; S1, 0.05% SGP; S2, 0.10% SGP; MO1, 0.05% MnO; MO2, 0.10% MnO; Mn1, 0.05% MnSO_4_; Mn2, 0.10% MnSO_4_; SMO, 0.05% SGP + 0.05% MnO; SMn, 0.05% SGP + 0.05% MnSO_4_. The data are presented as means ± SDs (*n* = 3). Different letters among each treatment indicate significant differences as determined by the LSD test (*p* < 0.05).

Figure 4 illustrates the effects of different treatments on Mn concentration in wheat organs. Soil application of Mn fertilizer alone or in combination with SGP generally increased Mn concentration in wheat organs, particularly in the MnSO_4_ and combined treatments (Figure 4). While most organs showed significant increases, the magnitude of these effects varied; for instance, no significant changes were observed in the panicle, NI, and IN1 under certain treatments. Specifically, compared to the control, applying MnSO_4_ and MnO alone increased Mn concentrations in grains by 35.9–76.2% and 39.9–59.2%, respectively. In contrast, applying SGP alone significantly decreased Mn concentrations in the nine organs by 39.5%—54.7% (Figure 4). Moreover, the combined application of SGP and Mn fertilizers resulted in Mn concentrations similar to, but slightly lower than, those of the single Mn treatments. The concentrations of Mn in grains from SMO and SMn treatments increased significantly by 59.3% and 27.0%, respectively, relative to the control treatment (Figure 4a).

Additionally, the correlation analysis of Mn concentrations among the nine wheat organs demonstrated distinct allocation patterns influenced by different amendments (Figure S2). In the single SGP treatment, grain Mn was only significantly correlated with panicle Mn (Figure S2a), while both Mn fertilizer treatments notably expanded the range of organs significantly related to Mn content in grains as compared to the single SGP treatment. Under single MnO treatment, grain Mn concentration showed significant positive correlations with Mn in the glume, flag leaf, stem (straw), other leaves, and root (Figure S2b). Furthermore, MnSO_4_ application resulted in significant positive correlations between grain Mn and Mn concentrations in all eight other organs except for node 1 (Figure S2c). In addition, co-application of SGP and Mn fertilizer resulted in a reduced number of organs exhibiting significant correlations with grain Mn concentration compared to the single Mn fertilizer treatment, but a great number of organs showed significant correlations relative to the single SGP treatment, primarily concentrating in the panicle, node 1, and stem (Figure S2d).

### 2.5. Correlation Between Cadmium and Manganese Concentrations in Wheat Organs

**Figure 5:**
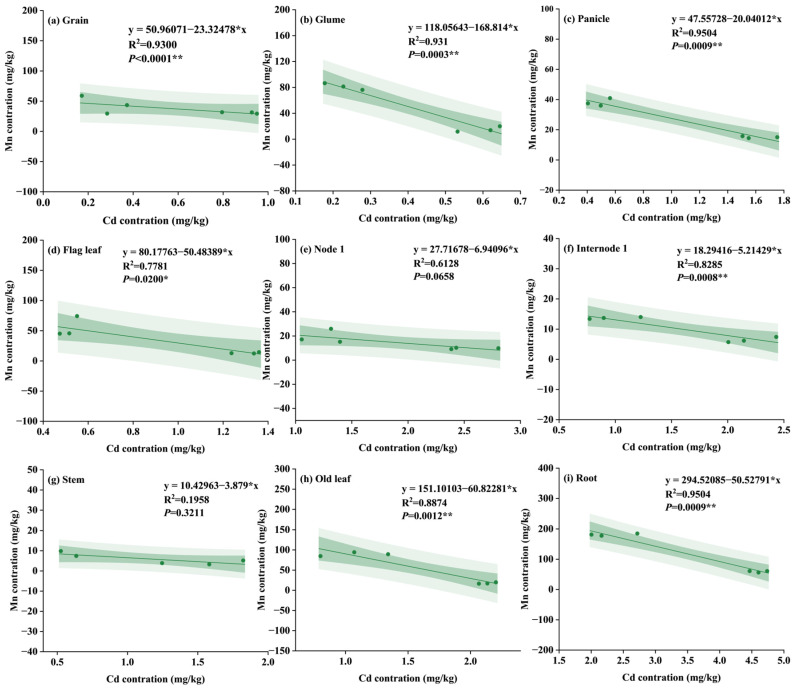
Correlation analysis between Cd and Mn concentrations across different wheat organs under the combined treatment of SGP and MnSO_4_: (**a**) grain, (**b**) glume, (**c**) panicle, (**d**) flag leaf, (**e**) internode 1, (**f**) node 1, (**g**) old leaf, (**h**) stem, and (**i**) root. Each point represents an individual replicate. The solid line represents the linear regression fit; the narrower shaded areas represent the 95% confidence intervals, while the wider shaded areas represent the 95% prediction intervals. The correlation coefficient (R) and significance level (*p*-value) for each organ are denoted. *, and ** indicate significance at the 0.05 and 0.01 probability levels, respectively.

The correlation between Mn and Cd concentrations in different wheat organs was analyzed under various treatments to elucidate the interaction between Cd and Mn in wheat. In general, the correlation between Cd and Mn was variable across different organs, but it highly depended on the specific plant organ and the type of amendment applied. As shown in Table 1 and Figure S3, single SGP treatment failed to establish systematic Cd-Mn antagonistic relationships across organs, showing only occasional positive or negative correlations in some organs. When MnO was applied alone, significant positive correlations between Cd and Mn were observed in older leaves and roots, indicating co-accumulation in these organs (Figure S4). In contrast, single MnSO_4_ treatment demonstrated a clearer Cd-Mn antagonistic trend. Significant negative correlations were found in grains and node 1, indicating effective suppression of Cd accumulation by Mn in these critical organs. Notably, a significant positive correlation persisted in roots, suggesting ineffective competition during root uptake (Figure S5). Critically, co-application of SGP and Mn fertilizer fundamentally transformed Cd/Mn relationships across organs. The combination of SGP and MnO (SMO) treatment established significant negative correlations in the grain, panicle, stem, older leaves, and root (Figure S6), while the combination of SGP and MnSO_4_ (SMn) treatment further enhanced this pattern, forming significant negative correlations in seven organs including the grain, glume, panicle, flag leaf, internode 1, older leaves, and root (Figure 5). Collectively, while these correlation findings (based on *n* = 6) suggest that the combined treatments promoted a systematic Cd/Mn antagonism throughout the plant, these results should be viewed as indicative of potential physiological trends rather than definitive causal mechanisms, particularly considering the statistical limitations of the current sample size.

### 2.6. Cd and Mn Transport Characteristics in Wheat Plants

As illustrated in Table 2, the addition of SGP and Mn fertilizers alone or in combination had substantial effects on the internal Cd transport characteristics in wheat plants. For Cd, single SGP treatment significantly reduced the root bioconcentration factor (BCF_Soil-R_) as compared to the control. Conversely, both Mn fertilizer treatments enhanced the root BCF. Moreover, the combined Mn fertilizers with SGP treatments (SMO and SMn) were most effective in reducing Cd uptake, lowering the root BCF by 44.8% and 54.9%, respectively, with values markedly lower than those of all other treatments. The TFs were determined to evaluate the ability of Cd/Mn transport within wheat organs. As shown in Table 2, soil application of 0.05% SGP (S1) significantly decreased Cd translocation factors from root to aerial organs (TF_R-N1_, TF_R-IN1_, TF_R-FL_, and TF_R-GR_) and from node 1 to grain (TF_N1-GR_) but significant increased the Cd translocation factor from flag leaf to grain (TF_R-IN1_) as compared to the control treatment. In contrast, the combined treatments (SMO and SMn) increased TF_R-N1_ and TF_R-IN1_ by 6.3–25.0% and 34.4–43.8%, respectively, as compared to the S1 treatment. However, they effectively reduced the critical transport from internode 1 to grain (TF_IN1-GR_) by 22.5–32.5% and from flag leaf to grain (TF_FL-GR_) by approximately 37.3% when compared to S1. For Mn, single SGP treatment reduced Mn translocation from root to grain (TF_R-GR_) by 46.2% and sharply decreased Mn translocation from node 1 and internode 1 to grain (TF_N1-GR_ and TF_IN1-GR_) by 54.4% and 61.6%, respectively. The combined treatments, particularly SMO treatment, induced the most dramatic shift, drastically reducing Mn transport from the root to all measured aerial organs (TF_R-N1_, TF_R-IN1_, TF_R-FL_, TF_R-GR_) by 73.1–81.3%, while simultaneously enhancing the transport from node 1 and internode 1 to grain (TF_N1-GR_ and TF_IN1-GR_) by 44.3% and 12.2%, respectively, relative to the control. Collectively, the combined application of SGP and Mn fertilizers, particularly the combination of SGP and MnSO_4_, not only reduced root Cd uptake from soil but also reconfigured the internal Cd transportation by suppressing Cd transfer from flag leaf and internode to the grain, ultimately leading to a decrease in grain Cd accumulation.

## 3. Discussion

The high immobilization efficiency of sulfhydryl-grafted palygorskite (SGP) in Cd-contaminated soil has been well-documented in numerous studies. The immobilization mechanism primarily involves the complexation of Cd through surface functional groups, with the thiol groups playing the dominant role [20]. According to Lewis acid–base theory, the sulfur (S) atom in thiol groups serves as an electron-pair donor, facilitating the formation of stable Cd-S coordination bonds through specific chemisorption [22]. Beyond direct complexation, SGP can alter the distribution of soil Cd fractions, typically transforming the highly bioavailable exchangeable and carbonate-bound fractions into less mobile fractions, thereby reducing Cd bioavailability [20]. The current study offers direct validation that SGP application considerably decreased the soil DTPA-extractable Cd concentration (Figure 1a), hence demonstrating its effective immobilization capacity. Therefore, in the combined application of SGP and manganese (Mn) fertilizers, SGP functions as the first line of defense by immobilizing Cd at the soil–root interface, thereby reducing its bioavailability and root exposure, as directly evidenced by the significantly decreased root BCF (Table 2).

In contrast to SGP, Mn fertilizers did not significantly affect soil Cd availability (Figure 1a) but still reduced grain Cd content, indicating that they function via plant physiological processes rather than soil chemistry. As divalent cations with similar chemical properties, Cd and Mn are known to compete for shared uptake and transport pathways, including the NRAMP and ZIP families [6]. Direct evidence for this Cd/Mn antagonism comes from the significant negative correlations between their concentrations in wheat organs under MnSO_4_ treatment (Table 1). The application of Mn fertilizers increases the ratio of Mn/Cd in the rhizosphere and plant organs, thereby leading to competition with Cd for these transporters. Beyond this direct competition, Mn application could also influence the expression of key genes involved in Cd transport. For instance, studies have shown that exogenous Mn can inhibit the expression of TaNramp5, a major transporter for Mn and Cd influx in roots [20]. Furthermore, Mn can downregulate the expression of efflux transporters TaHMA2 and TaLCT1, which are responsible for Cd loading into the xylem for root-to-shoot translocation, while upregulating the vacuolar sequestration gene TaHMA3 [20,30,31]. This reconfiguration of gene expression limits Cd translocation from root to shoot and promotes its sequestration in root vacuoles, collectively contributing to reduced grain Cd accumulation.

Notably, a distinct contrast in grain Cd reduction was observed between the two Mn fertilizers, with MnSO_4_ proving consistently more effective than MnO (Figure 3a). The superior performance of MnSO_4_ over MnO, particularly in establishing a more pervasive Cd/Mn antagonism, can be attributed to their fundamental differences in solubility and plant bioavailability. MnSO_4_ is highly water-soluble, providing readily available Mn ions in the soil solution. The rapid dissolution of MnSO_4_ ensures a quick and substantial supply of Mn to the plant, as evidenced by the generally higher Mn concentrations in wheat organs under MnSO_4_ treatment compared to MnO in the current study (Figure 4). Therefore, the rapid release of Mn from MnSO_4_ allows it to quickly enter the plant through the transport pathways shared with Cd, which could potentially inhibit Cd uptake by roots and its translocation to the shoot. Interestingly, despite the characteristically higher solubility of MnSO_4_, the observed greater concentration of DTPA-extractable Mn in the MnO-treated soil presents an apparent paradox (Figure 1). This discrepancy is primarily attributable to their distinct chemical behaviors in soil. The Mn ions derived from MnSO_4_, which are readily available to plant, are inherently more susceptible to leaching or rapid fixation by soil components after dissolution [32,33]. In contrast, the less soluble MnO might act as a slow-release fertilizer [34]. Consequently, MnO application can establish a stable Mn reservoir in soil, which consistently replenishes the soil available pool, thereby resulting in elevated level of total available Mn over time [35].

Thus, the combined application of SGP and Mn fertilizers represents a highly integrated remediation strategy. In this system, SGP primarily immobilizes Cd in the soil, directly reducing its bioavailability and consequently decreasing total Cd influx into plants (Table 2). Meanwhile, Mn fertilizers, particularly MnSO_4_, act through a restrictive mechanism and effectively limit Cd allocation to grains through competitive inhibition, ultimately reducing its final concentration. This combined mechanism is clearly evidenced by the translocation data showing that combined treatments enhance Cd translocation from root to shoot but dramatically suppress final allocation to grains (Table 2). This indicates that despite initial upward mobilization from the root, absorbed Cd becomes effectively retained in critical transfer organs (such as node 1 and internode 1) due to Mn competition, thereby reducing its transfer to the grain. Moreover, the efficient redistribution of Mn from these critical transfer organs to the grain in the combined treatments further confirms that Mn alters Cd distribution within wheat plants.

Thus, the combined application of SGP and Mn fertilizers represents a highly integrated remediation strategy, functioning as a multi-stage defense system. In this system, SGP acts as the first line of defense by immobilizing Cd in the soil, directly reducing its bioavailability and consequently decreasing total Cd influx into plants (Table 2). Meanwhile, Mn fertilizers, particularly MnSO_4_, provide a complementary defense through a restrictive mechanism that effectively limits Cd allocation to the grain through competitive inhibition. This dual-layered approach ensures a more integrated protection for wheat safety; even if environmental fluctuations in alkaline soils lead to a transient increase in Cd bioavailability, the internal antagonism from Mn consistently restricts Cd transfer to the grain.

Correlation analysis provides additional spatial insights into this combined mechanism. While Cd distribution showed strong correlations among organs under single SGP treatment (Figure S1a), single Mn application weakened these relationships (Figure S1b,c). Notably, the combined treatment restored the relationships among organs while establishing a new equilibrium with lower Cd and higher Mn levels (Figure S1d). The consistent negative relationship between Cd and Mn concentrations across multiple organs provides clear evidence that the interaction between these two elements is a competitive relationship within the plant. Future research should focus on validating this dual mechanism under field conditions across varying soil properties and climate, while molecular studies could further elucidate the temporal regulation of key transporter genes in response to combined treatment.

## 4. Materials and Methods

### 4.1. Soil, Wheat, and Immobilization Materials

A pot experiment was conducted in the Agro-Environmental Protection Institute, Ministry of Agriculture and Rural Affairs of China, from March to June 2023, to investigate the immobilization efficiency of different combinations of SGP and Mn fertilizers on Cd-contaminated alkaline soil. The soil utilized in pot experiment was a calcareous fluvo-aquic soil, obtained from the top layer (0–20 cm) of a wheat farmland located in Xinxiang, Henan Province. The basic physicochemical properties of soil were as follows: pH value 8.14, cation exchange capacity (CEC) 4.32 cmol/kg, organic matter 28.40 g/kg, total nitrogen 1.3 g/kg, available phosphorus 35.63 mg/kg, available potassium 909.30 mg/kg, total Cd 2.40 mg/kg, and total Mn 138.61 mg/kg. The detailed methods for soil physicochemical property determination are available in Supporting Information (SI) Text S1. After removing the visible plant residues and stones, the collected soils were air-dried, homogenized, ground, and filtered through a 2 mm sieve before use.

The spring wheat (*Triticum aestivum* L.) cultivar Ningchun4, acquired from the College of Agronomy, Inner Mongolia Agricultural University, was used in this experiment. SGP was synthesized in the laboratory using 3-mercaptopropyltrimethoxysilane and natural palygorskite via the high-speed shear method [36], and the specific synthesis procedure and the basic properties of SGP are described in Text S1 and Table S1. The two Mn fertilizers (manganese monoxide (MnO) and manganese sulfate (MnSO_4_)) used in the pot experiment were purchased from Macklin Inc. (Macklin, Shanghai, China).

### 4.2. Experimental Design and Sampling

In this study, nine different treatments were evaluated and designated as follows: CK (control), S1 and S2 (SGP at 0.05% and 0.10%, *w*/*w*), MO1 and MO2 (MnO at 0.05% and 0.10%, *w*/*w*), Mn1 and Mn2 (MnSO_4_ at 0.05% and 0.10%, *w*/*w*), SMO (0.05% SGP + 0.05% MnO, *w*/*w*), and SMn (0.05% SGP + 0.05% MnSO_4_, *w*/*w*). Each treatment was performed in triplicate for a total of 27 pots. For each pot, the tested soil was thoroughly mixed with amendments and solid basal fertilizers (0.2 g N/kg soil as CO(NH_2_)_2_, 0.15 g P_2_O_5_/kg as CaH_2_PO_4_ H_2_O, and 0.2 g K_2_O/kg as KCl) before being placed into plastic pots with 4 kg soil. A total of 35 wheat seeds were evenly sown in each pot, and the seedlings were thinned to 20 after 20 days. During the wheat growing season, soil moisture was maintained at 60–70% of field water capacity (FWC). This was achieved using the gravimetric method, where pots were weighed every 1–2 days and replenished with deionized water to compensate for evapotranspiration loss. This consistent watering schedule (every 2–5 days depending on growth stage) ensured a stable moisture environment for all treatments.

At the wheat maturity stage, the whole wheat plants and corresponding soil in each pot were sampled. After being harvested, wheat plants were divided into nine parts: root (R), stem (LS), old leaf (OL), flag leaf (FL), node 1 (N1), internode 1 (IN1), glume (G), panicle (P), and grain (WG). All wheat parts were then washed carefully with deionized water and dried at 70 °C in an oven to a constant weight. The dry weight of grains was recorded to determine the grain yield per pot. Finally, all parts were ground into powder. The soil samples were separated into two parts: one was immediately shock-frozen in liquid nitrogen and stored at −80 °C for microbial analysis, while the other was air-dried and passed through 20- and 100-mesh sieves for further analysis. To investigate the underlying microbial mechanisms, six representative treatments (CK, S1, MO1, Mn1, SMO and SMn) were selected for microbial community analysis.

### 4.3. Sample Analysis

#### 4.3.1. Determination of Total Cd and Mn Concentrations in Wheat Parts

Wheat plant samples (0.25 g) were digested with 8 mL HNO3 using a DigiBlock ED54 digestion system at 80 °C (1.5 h), 120 °C (1.5 h), 150 °C (3 h), and 170 °C (0.5–1.5 h) (DigiBlock ED54, LabTech, Beijing, China). The digested solutions were diluted to 50 mL with deionized water and filtered through a 0.45 μm filter membrane. The concentrations of Cd, Mn, Cu, Zn, Fe, and Mg in digested solutions were analyzed by inductively coupled plasma mass spectrometry (ICP-MS, iCAP Q, Thermo Fisher Scientific, Waltham, MA, USA). To access the analytical values of the wheat parts (QC/QA), standard wheat flour reference materials (GBW08503c, Academy of State Administration of Grain, SAAG) were treated and analyzed in a similar way. The recovery rate for GBW08503c was 85–105%.

#### 4.3.2. Determination of Soil Properties

Soil pH was measured in a ratio of soil to deionized water of 1:2.5 (*w*:*v*) using a pH meter (PB-10, Sartorius, Göttingen, Germany). The total concentrations of Cd and Mn in soil (0.25 g) were digested with 8 mL HNO_3_ and 4 mL hydrofluoric acid using a digestion system at 120 °C (1 h), 150 °C (2 h), and 170 °C (0.5–1 h). The concentrations of available metals in soil (5 g, 20 mesh) were extract ed by diethylenetriaminepentaacetic acid (DTPA) solution (0.005 M DTPA, 0.01 M CaCl_2_, and 0.1 M triethanolamine adjusted to pH 7.3 with HCl) following the extraction procedure of Lindsay and Norvell [37]. The speciation distribution of Cd in soil (1 g, 100 mesh) was analyzed following the sequential extraction procedures of [38,39] (as detailed in Text S1 and Table S2). This procedure partitions soil Cd into seven distinct fractions: (i) water-soluble + exchangeable (F1), (ii) carbonate-bound (F2), (iii) manganese oxide-bound (F3), (iv) amorphous iron oxide-bound (F4), (v) crystalline iron oxide-bound (F5), (vi) organically bound (F6), and (vii) residual (F7) fractions. The concentrations of Cd and Mn in soil digested solutions and extracted solutions were analyzed using ICP-MS after filtering through a 0.45 μm membrane filter.

#### 4.3.3. Soil Bacterial Community Analysis

The analysis of soil microbial community composition was performed using a high-throughput sequencing platform provided by Majorbio Bio-Pharm Technology Co., Ltd. (Shanghai, China). Total genomic DNA was extracted from 0.5 g of fresh soil using a MoBio PowerSoil^®^ DNA Isolation Kit (MoBio Laboratories, Carlsbad, CA, USA) according to the manufacturer’s instructions. DNA extraction quality was assessed by 1% agarose gel electrophoresis, while concentration and purity were determined using a NanoDrop 2000 spectrophotometer (Thermo Scientific, USA). The V3–V4 variable region of the 16S rRNA gene was amplified by PCR with primers 338F (5′-ACTCCTACGGGAGGCAGCAG-3′) and 806R (5′-GGACTACHVGGGTWTCTAAT-3′). The PCR thermal cycling conditions were: 95 °C for 3 min; followed by 27 cycles of 95 °C for 30 s, 55 °C for 30 s, and 72 °C for 45 s; with a final extension at 72 °C for 10 min. Following successful amplification, PCR products were quantified using a QuantiFluor™-ST system (Promega, Madison, WI, USA) and sequenced on an Illumina MiSeq PE300 platform. The raw sequence data were processed using the QIIME2 pipeline (version 2022.2). Quality filtering, denoising, and merging were performed using DADA2 to generate amplicon sequence variants (ASVs). Taxonomic assignment was conducted against the SILVA (release 138) database.

### 4.4. Calculation of Cd Translocation from Soil to Plant

The transfer characteristics of Cd from soil to plant organs at the mature stage of wheat were evaluated in terms of the bioconcentration factor (BCF) and transfer factor (TF), which were calculated as follows:

BCF = C root/Csoil(1)

TFi − j = Cj/Ci(2)

where Ci and Cj represent the Cd concentrations in the source organ and the sink organ, respectively (e.g., for TF_R-N1_, i is the root and j is node 1; for TF_N1-GR_, i is node 1 and j is the grain). Csoil represents the concentrations of total Cd in the treated soils after the wheat plants were harvested (mg/kg).

### 4.5. Statistical Analysis

Microsoft Excel 2010 and Origin 2019b software were used for data processing and figure drawing. The experimental data were expressed as three-replicate means ± standard deviations. One-way analysis of variance (ANOVA) was used to analyze significant differences among treatment groups, and the statistical significance level was set at *p* < 0.05 with a 95% confidence interval.

## 5. Conclusions

The present study clearly demonstrates that the combined application of SGP and Mn fertilizers, especially MnSO_4_, achieved a superior reduction in grain Cd concentration compared to the single SGP treatment. Simultaneously, this combined effect was accompanied by a significant increase in grain Mn content. Furthermore, soil microbial community analysis confirmed the environmental safety of this co-application, as it showed no substantial shifts in bacterial diversity or community structure. This combined effect is attributed to a dual mechanism: SGP immobilizes Cd in the soil, while Mn competes with and inhibits Cd transport within the plant. Future work should focus on field-scale validation and in-depth exploration of the molecular mechanisms underlying the Cd-Mn antagonism.

## Figures and Tables

**Table 1:** The correlation coefficients (R) between Cd and Mn concentrations in different wheat organs.

Organ	SGP	MnO	MnSO_4_	SMnO	SMn
Grain	0.8931 *	−0.2456	−0.8154 *	−0.8436 *	−0.9644 **
Glume	0.1817	−0.5615	−0.7915	−0.6514	−0.9649 **
Panicle	0.6810	−0.5153	−0.8722 *	−0.9242 **	−0.9749 **
Flag leaf	0.9306 **	−0.7376	−0.3447	−0.7528	−0.8821 *
Node 1	−0.7089	0.7447	−0.9035 *	0.8117 *	−0.7828
Internode 1	−0.8912 *	−0.0100	−0.4713	0.2328	−0.9102 **
Stem	−0.1311	−0.0447	−0.1356	−0.8279 *	−0.4425
Old leaf	−0.6590	0.9487 **	−0.7866	−0.9661 **	−0.9421 **
Root	−0.4734	0.8538 *	0.9379 **	−0.9851 **	−0.9749 **

Note: * and ** indicate significance at the 0.05 and 0.01 probability levels, respectively.

**Table 2:** The BCFs and TFs of Cd and Mn in wheat organs.

Element	Treatment	BCF_Soil-R_	TF_R-N1_	TF_R-IN1_	TF_R-FL_	TF_R-GR_	TF_N1-GR_	TF_IN1-GR_	TF_FL-GR_
Cd	CK	3.08 ± 0.091 ^b^	0.55 ± 0.053 ^ab^	0.48 ± 0.039 ^a^	0.28 ± 0.009 ^a^	0.19 ± 0.014 ^a^	0.35 ± 0.033 ^ab^	0.41 ± 0.057 ^a^	0.68 ± 0.031 ^b^
	0.05% SGP (S1)	2.48 ± 0.15 ^c^	0.48 ± 0.044 ^b^	0.32 ± 0.004 ^b^	0.13 ± 0.020 ^c^	0.13 ± 0.007 ^bc^	0.27 ± 0.017 ^c^	0.40 ± 0.018 ^a^	1.02 ± 0.100 ^a^
	0.05% MnO (MO1)	3.46 ± 0.22 ^a^	0.55 ± 0.0.063 ^ab^	0.38 ± 0.044 ^ab^	0.21 ± 0.041 ^abc^	0.16 ± 0.009 ^ab^	0.29 ± 0.022 ^bc^	0.42 ± 0.042 ^a^	0.77 ± 0.123 ^ab^
	0.05% MnSO_4_ (Mn1)	3.45 ± 0.024 ^a^	0.26 ± 0.006 ^c^	0.39 ± 0.036 ^ab^	0.18 ± 0.076 ^bc^	0.10 ± 0.007 ^c^	0.40 ± 0.035 ^a^	0.26 ± 0.037 ^b^	0.65 ± 0.098 ^b^
	0.05% SGP + 0.05% MnO (SMO)	1.70 ± 0.15 ^d^	0.51 ± 0.078 ^ab^	0.43 ± 0.071 ^ab^	0.23 ± 0.093 ^ab^	0.13 ± 0.014 ^bc^	0.26 ± 0.013 ^c^	0.31 ± 0.053 ^b^	0.64 ± 0.071 ^b^
	0.05% SGP + 0.05% MnSO_4_ (SMn)	1.39 ± 0.052 ^e^	0.60 ± 0.083 ^a^	0.46 ± 0.110 ^a^	0.25 ± 0.024 ^ab^	0.16 ± 0.024 ^bc^	0.27 ± 0.077 ^c^	0.35 ± 0.067 ^ab^	0.64 ± 0.038 ^b^
Mn	CK	-	0.16 ± 0.013 ^b^	0.11 ± 0.012 ^b^	0.22 ± 0.030 ^ab^	0.52 ± 0.03 ^a^	3.18 ± 0.254 ^a^	4.84 ± 0.520 ^a^	2.34 ± 0.288 ^a^
	0.05% SGP (S1)	-	0.20 ± 0.037 ^ab^	0.15 ± 0.0136 ^a^	0.19 ± 0.085 ^bc^	0.28 ± 0.038 ^b^	1.45 ± 0.441 ^b^	1.86 ± 0.330 ^c^	1.72 ± 0.681 ^ab^
	0.05% MnO (MO1)	-	0.24 ± 0.031 ^a^	0.07 ± 0.0205 ^c^	0.16 ± 0.020 ^bc^	0.29 ± 0.007 ^b^	1.21 ± 0.192 ^b^	4.65 ± 0.891 ^ab^	1.82 ± 0.287 ^abc^
	0.05% MnSO_4_ (Mn1)	-	0.09 ± 0.010 ^c^	0.05 ± 0.012 ^cd^	0.24 ± 0.091 ^ab^	0.25 ± 0.087 ^b^	2.81 ± 0.167 ^b^	4.97 ± 0.731 ^a^	1.05 ± 0.285 ^cd^
	0.05% SGP + 0.05% MnO (SMO)	-	0.03 ± 0.012 ^d^	0.03 ± 0.009 ^d^	0.10 ± 0.026 ^c^	0.14 ± 0.034 ^c^	4.59 ± 0.916 ^c^	5.43 ± 0.643 ^a^	1.45 ± 0.285 ^bcd^
	0.05% SGP + 0.05% MnSO_4_ (SMn)	-	0.11 ± 0.029 ^c^	0.08 ± 0.002 ^c^	0.33 ± 0.075 ^a^	0.24 ± 0.080 ^d^	2.11 ± 0.167 ^d^	3.22 ± 0.732 ^bc^	0.79 ± 0.438 ^d^

Note: BCF_Soil-R_ represents the bioconcentration factor from soil to root. TF_R-N1_, TF_R-IN1_, TF_R-FL_, and TF_R-GR_ represent the translocation factors from root to node 1, internode 1, flag leaf, and grain, respectively. TF_N-GR_, TF_IN1-GR_, and TF_FI-GR_ represent the translocation factors from node 1, internode 1, and flag leaf to the grain, respectively. The data are presented as means ± SDs (*n* = 3). Different letters among each treatment indicate significant differences as determined by the LSD test (*p* < 0.05).

## Data Availability

The data that support the findings of this study are available from the corresponding author upon reasonable request.

## Supplementary Materials

The following supporting information can be downloaded at: https://www.mdpi.com/article/10.3390/plants15040621/s1, Figure S1: Correlation analysis of Cd concentrations in various wheat organs under different treatments; Figure S2: Correlation analysis of Mn concentrations in various wheat organs under different treatments; Figure S3: Correlation analysis between Cd and Mn concentrations across different wheat organs under the treatment of SGP; Figure S4: Correlation analysis between Cd and Mn concentrations across different wheat organs under the treatment of MnO; Figure S5: Correlation analysis between Cd and Mn concentrations across different wheat organs under the treatment of MnSO_4_; Figure S6: Correlation analysis between Cd and Mn concentrations across different wheat organs under the combined treatment of SGP and MnO; Table S1: The basic physical and chemical of sulfhydryl-grafted palygorskite (SGP); Table S2: The sequential extraction procedure for soil Cd; Text S1: Analytical methods of soil properties.
