# Supplementary material for: Combined Effects of Sulfhydryl-Grafted Palygorskite and Manganese Fertilizers in Reducing Cadmium Accumulation in Wheat
Source: Plants (Basel). 2026 Feb 15;15(4):621. doi: 10.3390/plants15040621 (PMC12944629; doi:10.3390/plants15040621)
Supplement: Supplementary file 1 [file plants-15-00621-s001.zip › plants-4102398-supplementary.pdf]

Combined effects of thiol-functionalized palygorskite and manganese fertilizer reduces cadmium accumulation in wheat

Xingru Wang, Zhijun Liu, Xuefeng Liang, Xichao Sun, Yuebing Sun, Qingqing Huang

Supplementary Material

Figure and Table caption in supplementary material：

Text S1 Analytical methods of soil properties

Table S1 The basic physical and chemical of sulfhydryl-grafted palygorskite (SGP)

Table S2 The sequential extraction procedure for soil Cd

Figure S1. Correlation analysis of Cd concentration in various wheat tissues under different treatments, (a) SGP, (b)MnO, (c) $MnSO_4$, (d) SMO+SMn.

Figure S2. Correlation analysis of Mn concentration in various wheat tissues under different treatments, (a) SGP, (b)MnO, (c) $MnSO_4$, (d) SMO+SMn.

Figure S3. Correlation analysis between Cd and Mn concentration across different wheat tissues under the treatment of SGP: (a) grain, (b)glume, (c) panicle, (d) flag leaf, (e) internode1, (f) node 1, (g) old leaf, (h) stem, (i) root.

Figure S4. Correlation analysis between Cd and Mn concentration across different wheat tissues under the treatment of MnO : (a) grain, (b)glume, (c) panicle, (d) flag leaf, (e) internode1, (f) node 1, (g) old leaf, (h) stem, (i) root.

Figure S5. Correlation analysis between Cd and Mn concentration across different wheat tissues under the treatment of $MnSO_4$: (a) grain, (b)glume, (c) panicle, (d) flag leaf, (e) internode1, (f) node 1, (g) old leaf, (h) stem, (i) root.

Figure S6. Correlation analysis between Cd and Mn concentration across different wheat tissues under the combined treatment of SGP and MnO : (a) grain, (b)glume, (c) panicle, (d) flag leaf, (e) internode1, (f) node 1, (g) old leaf, (h) stem, (i) root.

Text S1 Analytical methods of soil properties and

(1) Basic physical and chemical of the tested soil

The basic physical and chemical of the tested soil were analyzed based on the methods of Lu (2000). The soil pH value was measured using a pH electrode (PB-10; Sartorius, Germany) at a ratio of soil to water of 1:2.5. The soil cation exchange capacity (CEC) was determined using the ammonium acetate exchange method followed by Kjeldahl distillation. The soil organic matter was measured according to the Walkley-Black wet digestion method. The total nitrogen in the soil was measured using the Kjeldahl method. The soil available phosphorus was determined by the ascorbic acid-ammonium molybdenum method. The soil available potassium was extracted with ammonium acetate and determined by flame photometry. The total concentration of Cd and Mn in the soil (0.2500 g) was digested in an electrothermal digester (DigiBlock ED54, LabTech, Beijing, China) using 8 mL HNO_3 (guaranteed reagent) and 4 mL hydrofluoric acid (HF) (guaranteed reagent) at 120 ℃ for 1.0 h, and 150 ℃ for 2.0 h, respectively. The digestion process was complete when the digested solutions were 1-2 mL in volume. The digested solutions were then diluted to 50 ml with deionized water, filtered, and analyzed with ICP-MS (iCAP Q; Thermo Fisher Scientific, MA, USA). For quality assurance, a soil reference material (SRM2586 from National Institute of Standards and Technology, NIST) and blanks were also digested under the same conditions, with recovery rates ranging from 80% to 120%. The results showed that the relative standard deviation (RSD) was less than 5%, the limit of detection (LOD) was 0.012 mg/kg, the limit of quantification (LOQ) was 0.040 mg/kg, and the recovery percentages ranged from 80% to 120%, indicating that the procedures used to determine heavy metals in soil were relatively accurate and reliable

(2) The specific synthesis procedure of SGP

Sulfhydryl-grafted palygorskite (SGP) was synthesized in the laboratory using 3-mercaptopropyltrimethoxysilane and natural palygorskite via the high-speed shear method. In briefly, 500 g of natural palygorskite was mixed with 10 L of deionized water at 25 °C, and then stirred at 10,000 rpm for 10 minutes with a disperser (T65 digital Ultra-Turrax, IKA, Staufen, Germany) to make an aqueous gel. After that, 500 g of 3-Mercaptopropyltrimethoxysilane was added to the gel and stirred at 10,000 rpm for 10 min. The gel was then collected, naturally dried, ground, and sieved (0.85 mm) for further use.

(3) Seven-step modified sequential extraction procedure

Soil samples (1.00 g) combined with different extraction reagents (See Table S2) on a rotator (28 ± 2 rpm) for the seven-step extraction. At the end of each step, post-extraction solutions and solids were separated by centrifugation at a centrifugal force of 4000×g for 10 min; and the post-extraction solutions were filtered with a 0.45 μm membrane and analyzed with ICP-MS. Furthermore, mass balance analysis was performed to verify the heavy metals concentration determined by the seven-step modified sequential extraction. The mass balance of heavy metals was done by comparing the total metal content in the samples determined by single-stage digestion with the sum of heavy metals determined by sequential extraction. The heavy metal balance was more than 90% in compliance.

.

Table S1 The basic physical and chemical of sulfhydryl-grafted palygorskite (SGP)

	Properties	Value
	pH (H_2O)	7.68±0.05
	CaO (%)	1.21
	MgO (%)	20.48
SGP	SiO_2 (%)	64.42
	Al_2O_3 (%)	10.43
	Fe_2O_3(%)	0.89
	Total Cd (mg/kg)	0.12±0.03

Table S2 The sequential extraction procedure for soil Cd

Step	Speciation	Extractant	Conditoins
1	Water-soluble + Exchangeable (F1)	1 mol·L^{-1} Ammonium Acetate (pH 7.00)	Room temperature shaking for 24 h
2	Carbonate-bound (F2)	1 mol·L^{-1} Ammonium Acetate (pH 6.00)	Room temperature shaking for 24 h
3	Manganese oxide-bound (F3)	0.1 mol·L^{-1} Hydroxylamine Hydrochloride + 1 mol·L^{-1} Ammonium Acetate (pH 6)	Room temperature shaking for 0.5 h
4	Amorphous iron oxide-bound (F4)	0.1 mol·L^{-1} Ammonium Oxalate Buffer (pH 3.25)	Room temperature shaking for 4 h
5	Crystalline iron oxide-bound (F5)	0.1 mol·L^{-1} Ascorbic Acid Solution + 0.2 mol·L^{-1} Ammonium Oxalate Buffer (pH 3.25)	Water bath at 96 °C for 0.5 h
6	Organically bound (F6)	3 mL 0.02 mol·L^{-1} Acetic Acid, 5 mL 30% Hydrogen Peroxide, + 5 mL 30% Hydrogen Peroxide + 5 mL 3.2 mol·L^{-1} Ammonium Acetate (20% Nitric Acid)	Water bath at 85 °C for 2 h, Water bath at 85 °C for 3 h, Room temperature shaking for 0.5 h
7	Residual (F7)	Aqua Regia	Microwave Digestion

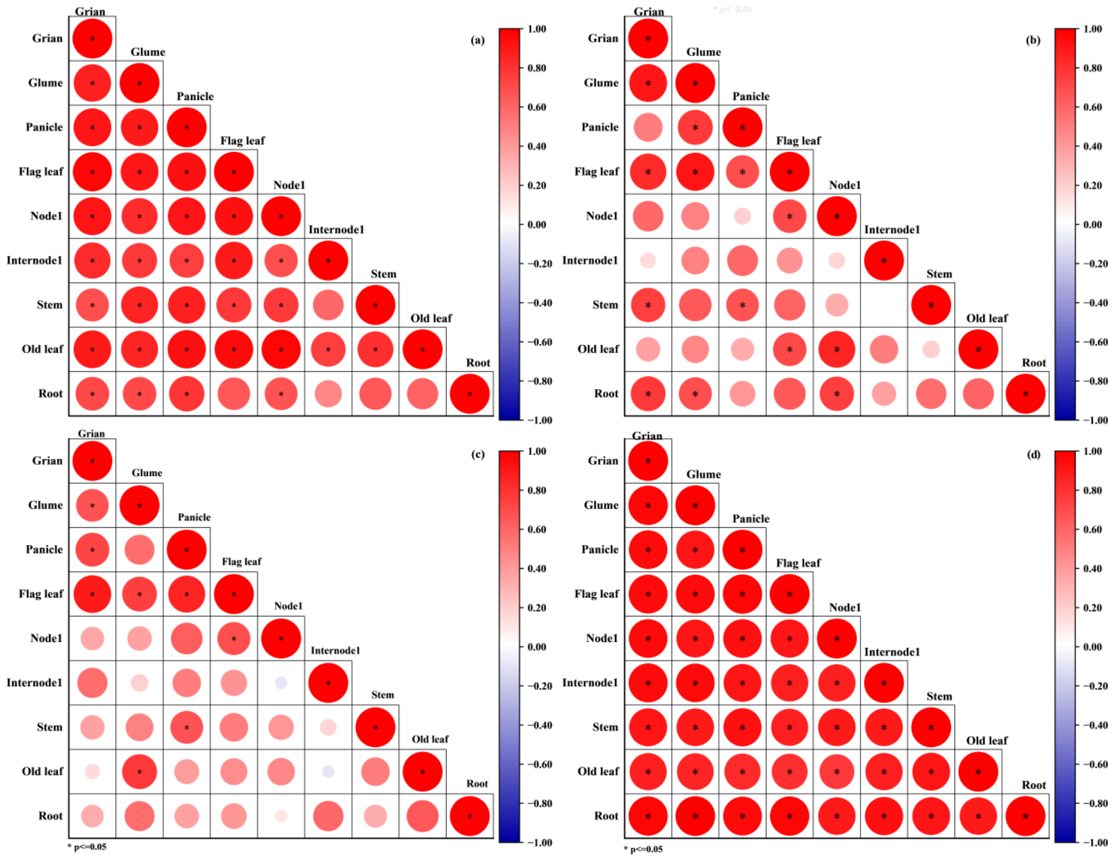

Figure S1 Correlation analysis of Cd concentration in various wheat tissues under different treatments, (a) SGP, (b)MnO, (c) $MnSO_4$, (d) SMO+SMn.

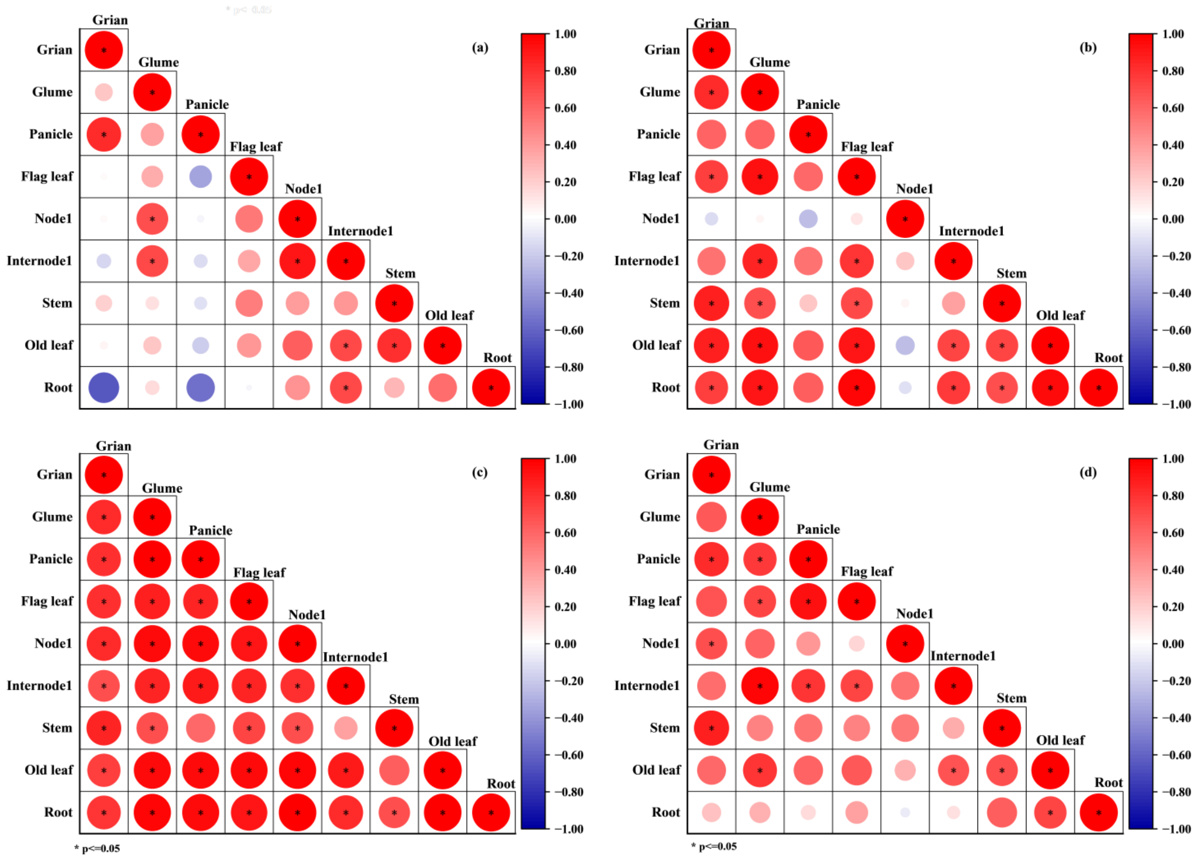

Figure S2 Correlation analysis of Mn concentration in various wheat tissues under different treatments, (a) SGP, (b)MnO, (c) $MnSO_4$, (d) SMO+SMn.

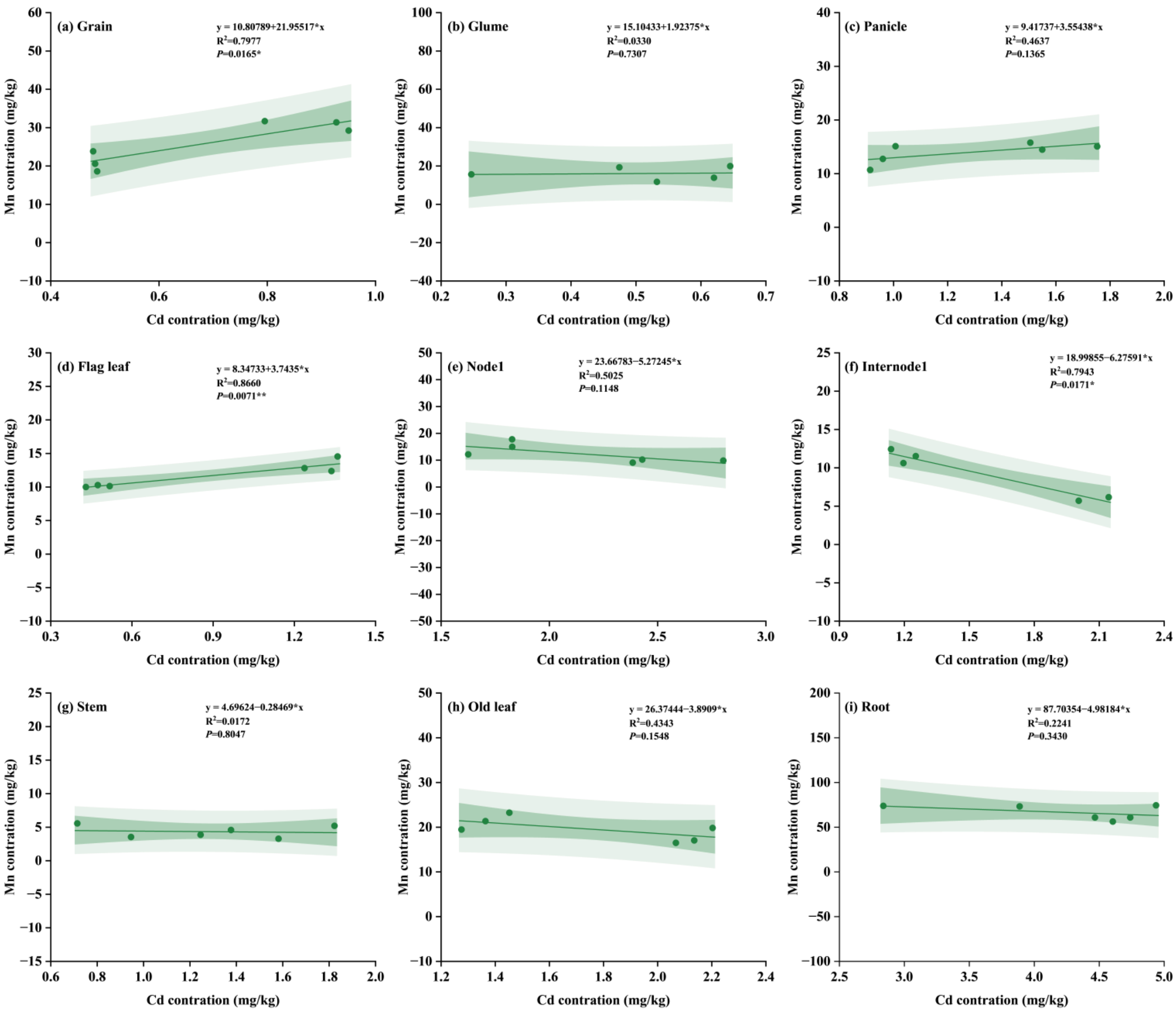

Figure S3. Correlation analysis between Cd and Mn concentration across different wheat tissues under the treatment of SGP: (a) grain, (b)glume, (c) panicle, (d) flag leaf, (e) internode1, (f) node 1, (g) old leaf, (h) stem, (i) root. Each point represents an individual replicate. The solid line represents the linear regression fit; the narrower shaded areas represent the 95% confidence intervals, while the wider shaded areas represent the 95% prediction intervals. The correlation coefficient (R) and significance level (*P*-value) for each tissue are denoted. * and ** indicate significance at the 0.05 and 0.01, probability levels, respectively.

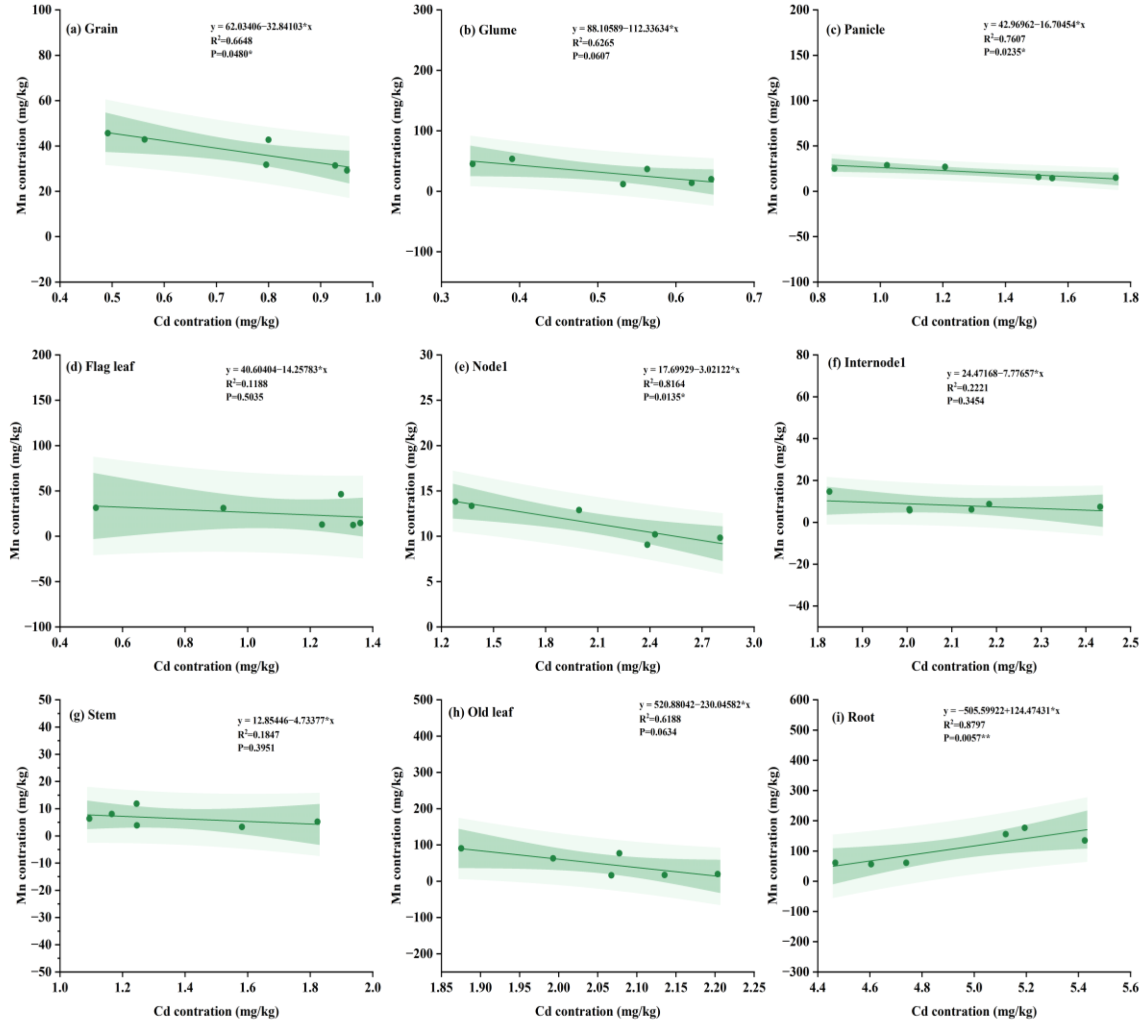

Figure S4. Correlation analysis between Cd and Mn concentration across different wheat tissues under the treatment of MnO : (a) grain, (b)glume, (c) panicle, (d) flag leaf, (e) internode1, (f) node 1, (g) old leaf, (h) stem, (i) root. Each point represents an individual replicate. The solid line represents the linear regression fit; the narrower shaded areas represent the 95% confidence intervals, while the wider shaded areas represent the 95% prediction intervals. The correlation coefficient (R) and significance level (*P*-value) for each tissue are denoted. * and ** indicate significance at the 0.05 and 0.01, probability levels, respectively.

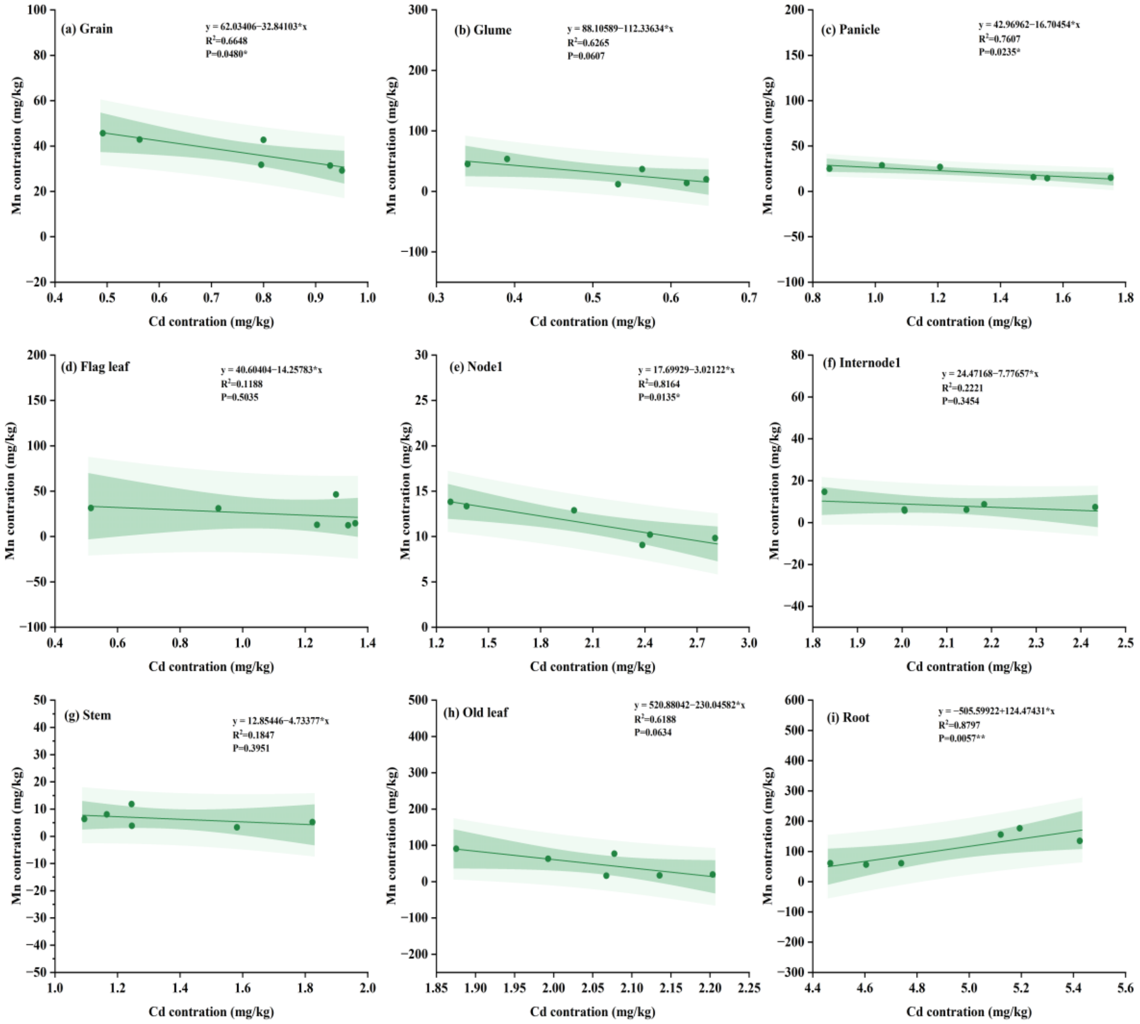

Figure S5. Correlation between Cd and Mn concentrations in various tissues of spring wheat under the treatments of $MnSO_4$ amendment: (a) grain, (b)glume, (c) panicle, (d) flag leaf, (e) internode1, (f) node 1, (g) old leaf, (h) stem, (i) root. Each point represents an individual replicate. The solid line represents the linear regression fit; the narrower shaded areas represent the 95% confidence intervals, while the wider shaded areas represent the 95% prediction intervals. The correlation coefficient (R) and significance level (*P*-value) for each tissue are denoted. * and ** indicate significance at the 0.05 and 0.01, probability levels, respectively.

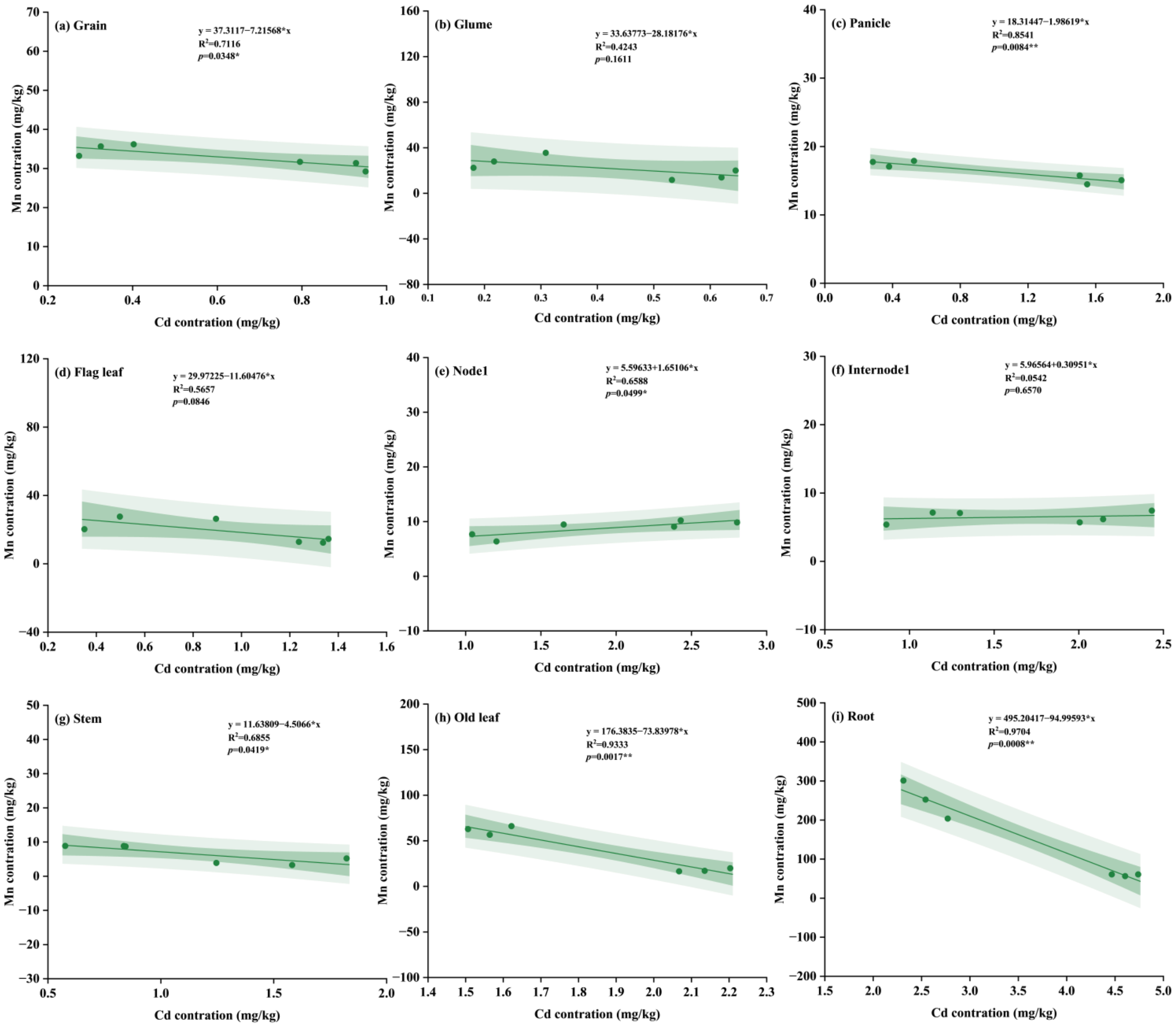

Figure S6. Correlation analysis between Cd and Mn concentration across different wheat tissues under the combined treatment of SGP and MnO: (a) grain, (b)glume, (c) panicle, (d) flag leaf, (e) internode1, (f) node 1, (g) old leaf, (h) stem, (i) root. Each point represents an individual replicate. The solid line represents the linear regression fit, with the shaded band indicating the 95% confidence interval. The correlation coefficient (R) and significance level (l (*P*-value)) for each tissue are denoted. *, **, and *** indicate significance at the 0.05, 0.01, and 0.001 probability levels, respectively.
